# Oxyresveratrol Inhibits IL-1β-Induced Inflammation via Suppressing AKT and ERK1/2 Activation in Human Microglia, HMC3

**DOI:** 10.3390/ijms21176054

**Published:** 2020-08-22

**Authors:** Phateep Hankittichai, Hua Jane Lou, Nitwara Wikan, Duncan R. Smith, Saranyapin Potikanond, Wutigri Nimlamool

**Affiliations:** 1 Department of Pharmacology, Faculty of Medicine, Chiang Mai University, Chiang Mai 50200, Thailand; phateep.han18@gmail.com (P.H.); saranyapin.p@cmu.ac.th (S.P.); 2 Graduate School, Chiang Mai University, Chiang Mai 50200, Thailand; 3 Department of Pharmacology, Yale University School of Medicine, New Haven, CT 06520, USA; jane.lou@yale.edu; 4 Institute of Molecular Biosciences, Mahidol University, Salaya, Nakorn Pathom 73170, Thailand; nitwara.wik@mahidol.edu (N.W.); duncan_r_smith@hotmail.com (D.R.S.)

**Keywords:** anti-inflammation, human microglia, interleukin-1β, oxyresveratrol, AKT activation, ERK1/2 MAPK activation

## Abstract

Oxyresveratrol (OXY), a major phytochemical component derived from several plants, has been proved to have several pharmacological properties. However, the role of OXY in regulating neuroinflammation is still unclear. Here, we focused mainly on the anti-neuroinflammatory effects at the cellular level of OXY in the interleukin-1 beta (IL-1β)-stimulated HMC3 human microglial cell line. We demonstrated that OXY strongly decreased the release of IL-6 and MCP-1 from HMC3 cells stimulated with IL-1β. Nevertheless, IL-1β could not induce the secretion of TNF-α and CXCL10 in this specific cell line, and that OXY did not have any effects on reducing the basal level of these cytokines in the sample culture supernatants. The densitometry analysis of immunoreactive bands from Western blot clearly indicated that IL-1β does not trigger the nuclear factor-kappa B (NF-κB) signaling. We discovered that OXY exerted its anti-inflammatory role in IL-1β-induced HMC3 cells by suppressing IL-1β-induced activation of the PI3K/AKT/p70S6K pathway. Explicitly, the presence of OXY for only 4 h could strongly inhibit AKT phosphorylation. In addition, OXY had moderate effects on inhibiting the activation of ERK1/2. Results from immunofluorescence study further confirmed that OXY inhibited the phosphorylation of AKT and ERK1/2 MAPK upon IL-1β stimulation in individual cells. These findings suggest that the possible anti-inflammatory mechanisms of OXY in IL-1β-induced HMC3 cells are mainly through its ability to suppress the PI3K/AKT/p70S6K and ERK1/2 MAPK signal transduction cascades. In conclusion, our study provided accumulated data that OXY is able to suppress IL-1β stimulation signaling in human microglial cells, and we believe that OXY could be a probable pharmacologic agent for altering microglial function in the treatment of neuroinflammation.

## 1. Introduction

In recent years, there has been accumulating information on inflammatory processes involving the etiopathology of neurological disorders [1]. Neuroinflammation is contributed by leukocytes, which invade the central nervous system (CNS) parenchyma, and a significant loss of blood–brain barrier (BBB) stability leading to advances in the development of several different neurodegenerative conditions which include Alzheimer’s disease, Parkinson’s disease, and multiple sclerosis [2]. Interestingly, much has been discovered about the association of increased interleukin-1 beta (IL-1β) and pro-inflammatory cytokine levels in the brain during inflammation [3]. Furthermore, the secretion of IL-1β results from the canonical “Nod-like” receptor family, pyrin domain containing 3 (NLRP3)-caspase-1 inflammasome in microglial-mediated neuroinflammation through cell-surface pattern recognition receptors (PRRs) recognized by the Toll/IL-1R family leading to CNS-resident microglia activation [4].

After IL-1β binds to its specific receptor, the initial downstream response is the recruitment of adaptor protein, myeloid differentiation protein 88 (MyD88), and the IL-1R-associated protein kinases (IRAKs) and TNF-receptor associated factor 6 (TRAF6) [5]. The complex MyD88–IRAK–TRAF6 signaling module can further trigger the signaling cascades by activating the nuclear factor-kappa B (NF-κB), the extracellular signal-regulated kinases 1/2 (ERK1/2), c-Jun-N-terminal kinases (JNKs), and p38 MAPKs, and the phosphatidylinositol 3-kinase (PI3K)/AKT [4,6]. Moreover, the IL-1β signaling can promote the synthesis and release of pro-inflammatory mediators such as interleukin 6 (IL-6), tumor necrosis factor α (TNF-α), monocyte chemoattractant protein-1 (MCP-1), and interferon-γ inducible protein 10 (CXCL10) from the immune cells [7,8]. However, the overproduction of such mediators is neurotoxic and probably interferes with neuronal function. Thus, the modulation of IL-1β can be particularly beneficial to attenuate neuroinflammatory response. Intriguingly, natural polyphenolic constituents have emerged as good candidates for the treatment of neuroinflammatory conditions [9].

Oxyresveratrol (*trans*-2,3′,4,5′-tetrahydroxystilbene, OXY) is a polyphenolic molecule, and it can be extracted from different plants, including *Artocarpus lakoocha* (*A. lakoocha*) [10,11]. Furthermore, OXY exerts several pharmacological activities, especially anti-inflammatory effects. Our previous study revealed that *A. lakoocha* extract (containing OXY as a major compound) decreased the lipopolysaccharide (LPS)-mediated release of nitric oxide (NO), MCP-1, IL-6, and TNF-α in RAW 264.7 macrophages by suppressing the phosphorylation of PI3K/AKT and the activation of NF-κB signal transduction pathway [10]. Interestingly, OXY has been reported to pass the blood–brain barrier in rats, exhibit neuroprotective effects in SH-SY5Y cells against hydrogen peroxide (H_2_O_2_) induction, ameliorate NLRP3 inflammasome activation, and block nuclear translocation of NF-κB molecule in rat cortical neuronal cells in response to glucose or oxygen deprivation [12,13,14]. Moreover, OXY presents its neuroprotective ability by counteracting reactive oxygen species (ROS) in activated mouse murine N9 microglia [15]. However, evidence for whether OXY inhibits IL-1β-induced inflammatory signaling in human microglia is still unexplored. Thus, we would like to determine the anti-inflammatory activities of OXY in human microglia stimulated by IL-1β.

Our study provided collected evidence that OXY can decrease IL-1β-mediated secretion of IL-6 and MCP-1 but not TNF-α and CXCL10 in HMC3 cells, in part through suppressing (PI3K)/AKT and ERK1/2 activation in response to IL-1β stimulation. As OXY reveals the ability to regulate inflammatory response in human microglial cells induced by IL-1β, we believe that OXY could be an alternative candidate for development as a promising agent with anti-neuroinflammatory properties.

## 2. Results

### 2.1. Effects of Oxyresveratrol (OXY) on the Viability of HMC3 Cells

**Figure 1:**
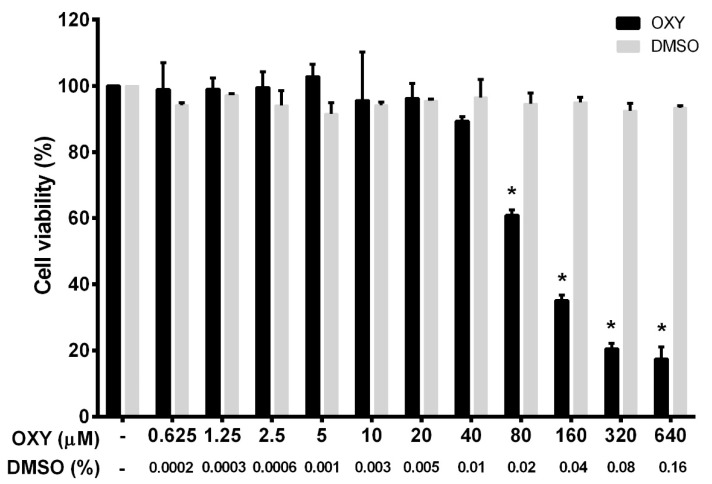
Effects of oxyresveratrol (OXY) on cell viability in HMC3 cells. The bars indicate percent cell viability of cells treated with OXY with the indicated concentrations (ranging from 0 to 640 μM) for 48 h and subjected to 3-(4,5-dimethylthiazol-2-yl)-2,5-diphenyltetrazolium bromide (MTT) assay. Data are represented as mean ± SD of three independent experiments; * *p* < 0.05 (compared to the untreated group).

To ensure that the tested concentrations were not toxic to the cells, we first performed a cell viability experiment for HMC3 incubated with OXY. OXY at various concentrations (0 to 640 μM) was added to the culture HMC3 over the course of 48 h before being subjected to a cell viability assay using MTT (3-(4,5-dimethylthiazol-2-yl)-2,5-diphenyltetrazolium bromide) reagent. The results, as seen in Figure 1, demonstrated that OXY was toxic to HMC3 cells when the concentration was increased, but those concentrations were evaluated to be 80 μM and above. Other concentrations of OXY lower than 80 μM proved to have no cytotoxicity to HMC3 cells. The pattern of dose-response cytotoxicity to OXY could be seen, with 93.5 μM evaluated to be its IC_50_. OXY at 640 μM revealed maximum cytotoxic effect, where more than 80% reduction of HMC3 cells was examined. However, OXY at concentrations under 40 μM did not alter the viability of HMC3 cells. DMSO, used as a vehicle control, at all concentrations (0% to 0.16%) related to those of DMSO concentrations in the OXY-treated group represented no obvious cytotoxic effect on HMC3 cells. To investigate anti-inflammatory activities of OXY on HMC3 cells, we chose OXY at 10, 20, and 40 μM, which were all nontoxic to the cell. These concentrations were used in every experiment of this study.

### 2.2. OXY Reduces Inflammatory Cytokine and Chemokine Secretion in IL-1β-stimulated HMC3 Cells

**Figure 2:**
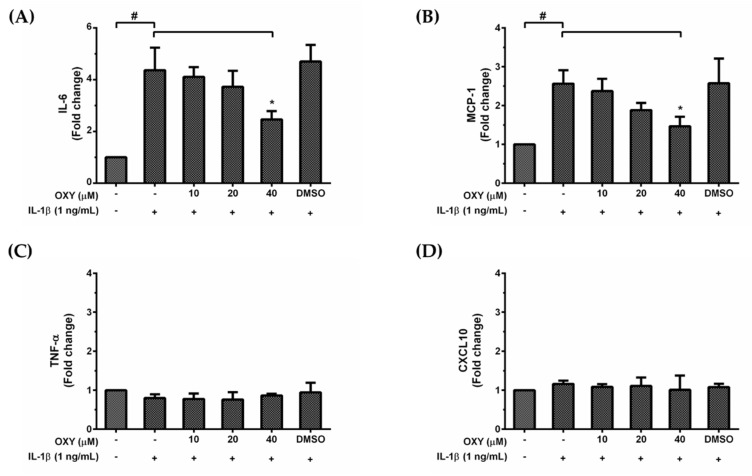
The roles of OXY on IL-1β-stimulated production of IL-6 (**A**), MCP-1 (**B**), TNF-α (**C**), and CXCL10 (**D**) in the supernatants of HMC3 cells pretreated with OXY at the different concentrations of 10, 20, and 40 μM for 4 h, and then induced with IL-1β (1 ng/mL) for 24 h. The bars indicate the secreted fold change of pro-inflammatory mediators which were determined by ELISA. Data are represented as mean ± SD of three independent experiments; ^#^ *p* < 0.05 (compared to the untreated group) or * *p* < 0.05 (compared to the IL-1β -treated group).

HMC3 cells are unique myeloid cells working in the brain as an important component of the immune defense system within the CNS. Since over production of inflammatory mediators can lead to neurotoxicity, we asked whether OXY treatment could modulate the inflammatory response to IL-1β activation. We used the ELISA technique to evaluate the amount of IL-6, TNF-α, MCP-1, and CXCL10 secreted in the culture supernatants of HMC3 cells treated with various concentrations of OXY and induced with IL-1β for 24 h. Interestingly, we found that IL-1β could markedly stimulate IL-6 and MCP-1 (Figure 2A,B) but not TNF-α and CXCL10 (Figure 2C,D) production in HMC3 cells. These inflammatory effects of IL-1β were reduced by OXY in a concentration-dependent manner. These results clearly indicate that OXY can alleviate the production of important inflammatory mediators in IL-1β-induced HMC3 cells. Although HMC3 cells have been reported to have no nitric oxide (NO) generation since there is no expression of inducible nitric oxide synthase (iNOS) [16], we still tested for the production of NO and the expression of iNOS in response to IL-1β stimulation and asked whether OXY has inhibitory effects on iNOS expression and NO production. As expected, data clearly showed that there was no iNOS expression and very low levels of NO in HMC3 induced with IL-1β, and OXY at all concentrations did not generate any difference in the level of iNOS and NO (Supplementary Materials Figure S1A–G).

### 2.3. IL-1β Does Not Stimulate Nuclear Factor Kappa B (NF-κB) Activation in HMC3 Cells, and OXY Does Not Decrease the Basal Production of Some Cytokines

**Figure 3:**
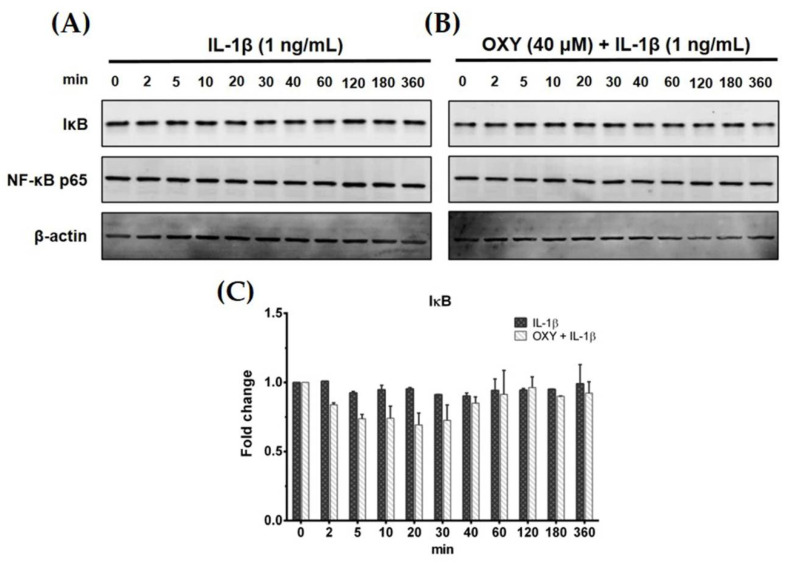
(**A**) Effects of OXY on the expression level of IκB in HMC3 cells induced by IL-1β for various time points (0–360 min); (**B**) the effects of OXY on the expression level of IκB after IL-1β stimulation for various time points. NF-κB p65 subunit was detected and used as an internal control; (**C**) quantitative analysis for IκB expression. Data are represented as mean ± SD of three independent experiments.

Generally, IL-1β can activate NF-κB activation in some human microglial cell lines. Therefore, to test our hypotheses whether IL-1β can upregulate the production of IL-6 and MCP-1 in HMC3 cells by triggering the NF-κB signaling pathway, and OXY may reduce this event, we used Western blot analysis to measure the degradation status of IκB after IL-1β activation. Interestingly, IL-1β treatment at 1 ng/mL for various time points (0–360 min) did not induce degradation of IκB (Figure 3A,C). Additionally, HMC3 cells pretreated with OXY at 40 μM for 4 h before IL-1β stimulation showed no difference in the downregulation of IκB in comparison to the untreated cells (Figure 3B,C). These data clearly showed that IL-1β does not stimulate NF-κB activation, and OXY does not have any effect on the degradation status of IκB. It suggests that OXY may inhibit IL-6 and MCP-1 production via modulating other signaling transduction pathways. One possible way is that OXY, which is well known to possess potent antioxidant effects, may reduce the production of intracellular reactive oxygen species (ROS) in the cells induced with IL-1β which may consequently suppress the inflammatory response of HMC3 upon IL-1β stimulation. To test this hypothesis, we performed an experiment to measure the intracellular level of ROS in the IL-1β-induced HMC3. Results clearly showed that IL-1β did not significantly induce ROS production in HMC3 cells (Supplementary Materials Figure S2A). However, we designed an experiment to confirm that this particular microglial cell line can generate intracellular ROS in response to hydrogen peroxide (H_2_O_2_). We found that H_2_O_2_ strongly induced ROS production in individual HMC3 cells, but OXY at all concentrations potently inhibited intracellular ROS in these cells (Supplementary Materials Figure S2A,B). The observations that IL-1β did not significantly induce ROS production in HMC3 cells, but OXY could reduce IL-1β-stimulated cytokine production, suggest that OXY may exert its anti-inflammatory effects mainly through manipulating specific signal transduction pathway.

### 2.4. OXY Inhibits PI3K/AKT and ERK1/2 Activation in HMC3 Upon IL-1β Stimulation

**Figure 4:**
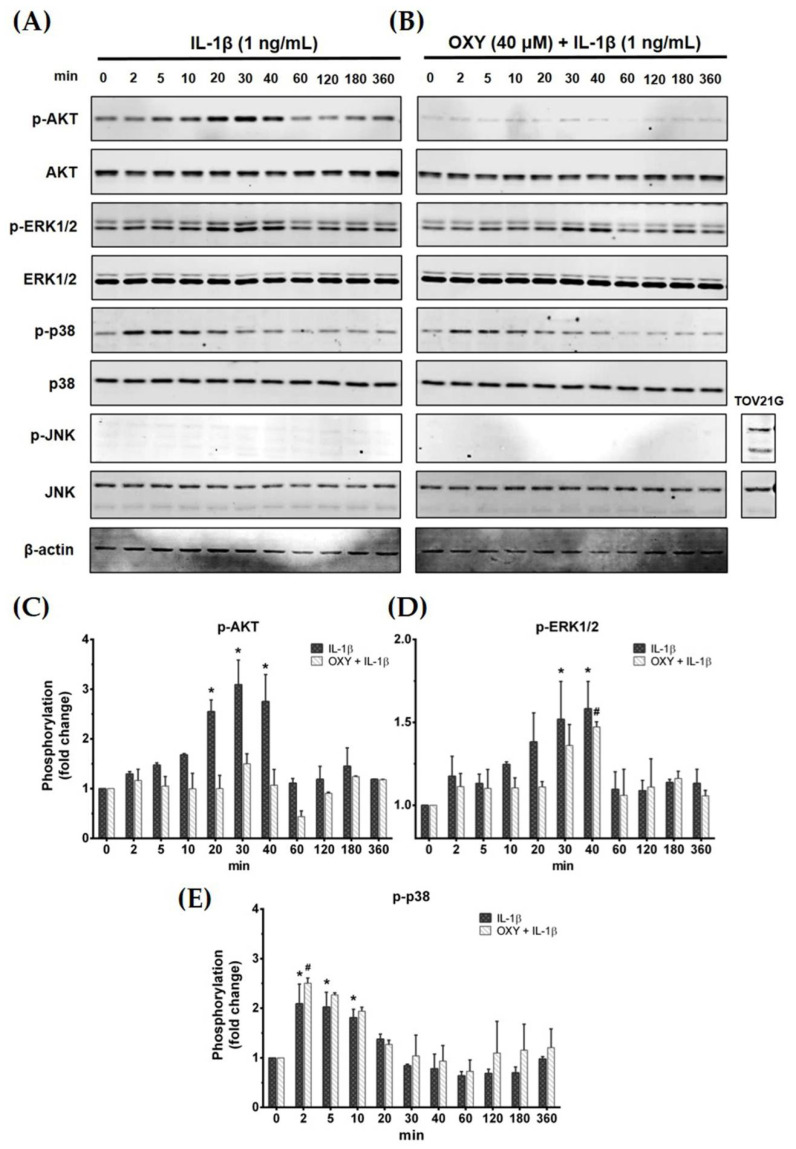
(**A**) The phosphorylation status of PI3K/AKT kinase and MAPKs, including ERK1/2, p38, and JNK, of IL-1β-induced HMC3 cells at 0 to 360 min; (**B**) the effects of OXY on suppressing the phosphorylation of AKT, ERK1/2, p38, and JNK after IL-1β activation; total proteins were detected and used as an internal control; (**C**) quantitative analysis for phosphorylated AKT (Ser473); (**D**) quantitative analysis for phosphorylated ERK1/2; (**E**) quantitative analysis for phosphorylated p38. Data are represented as mean ± SD of three independent experiments; ^#^ *p* < 0.05 (compared to the IL-1β-treated group after 0 min induction) or * *p* < 0.05 (compared to the OXY + IL-1β-treated group after 0 min).

Activation of the IL-1β receptor by IL-1β has been reported to induce the growth and survival signal transduction pathways [17]. Thus, we desired to investigate how OXY modulates the production of inflammatory cytokines in HMC3 in response to IL-1β stimulation. The early phosphorylation status of AKT and MAPK was examined using Western blotting in cells which were pretreated with OXY before the addition of IL-1β. As expected, we found that OXY drastically inhibited IL-1β-evoked phosphorylation of AKT (Ser473) at all time points over 12 h in HMC3 cells (Figure 4A–C). Furthermore, the phosphorylation of ERK1/2 was moderately suppressed by OXY treatment, whereas p38 and JNK phosphorylation status was not affected (Figure 4A,B,D,E). Surprisingly, we found that IL-1β did not activate the phosphorylation of JNK in HMC3 cells when compared with TOV21G, a human ovarian cancer cell line (used as a control for JNK phosphorylation), induced by 10% fetal bovine serum (FBS) for 30 min (Figure 4B).

**Figure 5:**
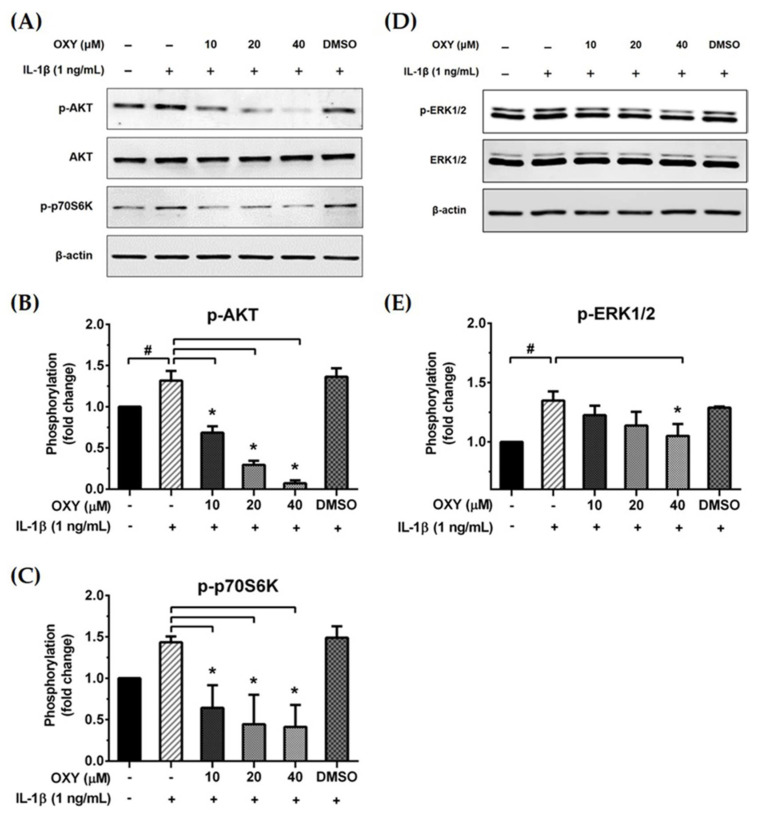
(**A** and **D**) Western blot analysis for the inhibitory effects of OXY on the phosphorylation of AKT, p70S6K, and ERK1/2 in cells treated with various concentrations of OXY (10, 20, and 40 μM) for 4 h before stimulation with IL-1β for 20 min; (**B**) quantitative analysis for AKT phosphorylation at Ser473; (**C**) quantitative analysis for p70S6K phosphorylation; (**E**) quantitative analysis for ERK1/2 phosphorylation. Total AKT, total ERK1/2, and beta actin (β-actin) were detected and used as an internal control. Data are represented as mean ± SD of three independent experiments; ^#^ *p* < 0.05 (compared to the untreated group) or * *p* < 0.05 (compared to the IL-1β-treated group).

**Figure 6:**
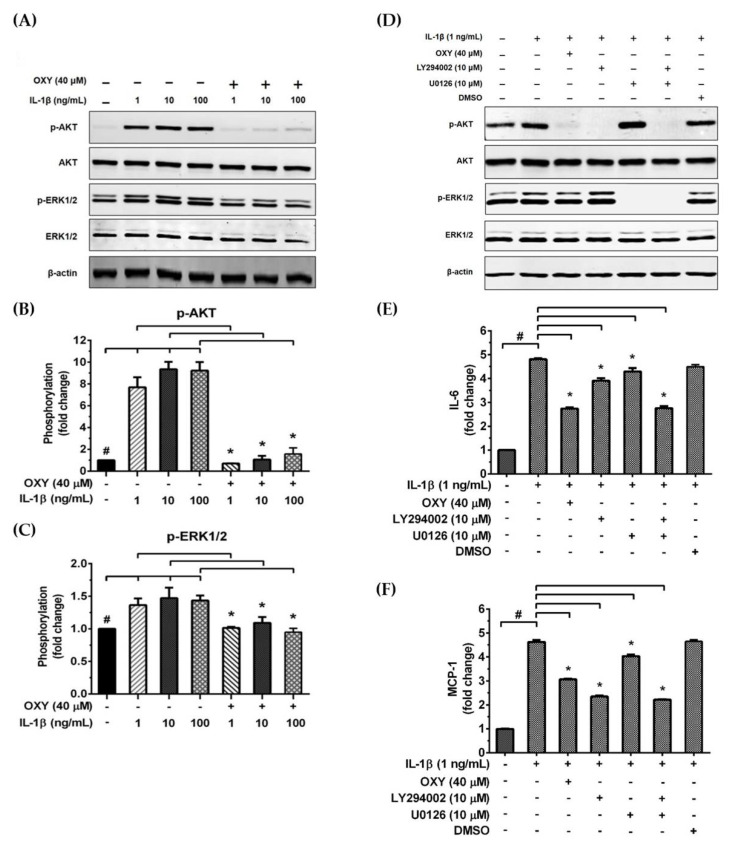
(**A**) Western blot analysis for the inhibitory effects of OXY on AKT and ERK1/2 phosphorylation in HMC3 after being treated with OXY (40 μM) for 4 h before stimulation with various concentrations of IL-1β (1, 10, and 100 ng/mL) for 20 min; (**B**) quantitative analysis for AKT phosphorylation at Ser473; (**C**) quantitative analysis for ERK1/2 phosphorylation. Total AKT and total ERK1/2 were detected and used as an internal control. (**D**) Western blot analysis detecting the phosphorylation status of AKT and ERK1/2 in response to IL-1β (1 ng/mL) for 20 min with or without the presence of 10 μM of LY294002 or 10 μM of U0126. The level of IL-6 (**E**) or MCP-1 (**F**) in the culture supernatants of HMC3 cells stimulated with IL-1β, with or without the presence of LY294002 or U0126. Data are represented as mean ± SD of three independent experiments; ^#^ *p* < 0.05 (compared to the untreated group) or * *p* < 0.05 (compared to the IL-1β-treated groups).

We further examined the early intracellular signaling by pretreatment of cells with OXY at the three different concentrations for 4 h before IL-1β stimulation. We discovered that OXY significantly inhibited phosphorylation of AKT at Ser473 and the isoform of p70 S6 kinase (p70S6K), which is a crucial target downstream to the PI3K/AKT pathway upon activation by IL-1β (Figure 5A–C). Notably, this inhibition of AKT phosphorylation and p70S6K by OXY was seen to be in a concentration-dependent manner. For the MAPK pathway, it revealed that the levels of ERK1/2 phosphorylation were slightly increased in the cells treated with IL-1β. However, this effect was potentially suppressed by pretreatment of cells with OXY (Figure 5D,E). Additionally, we investigated the effects of OXY on inhibiting AKT and ERK1/2 phosphorylation that was stimulated with increased concentrations of IL-1β in HMC3 cells. After cell were treated with IL-1β (1, 10, 100 ng/mL) for 30 min, phosphorylations of AKT and ERK1/2 were significantly increased (Figure 6A–C). However, the levels of phosphorylation of these kinases were drastically decreased in cells with OXY pretreatment for 4 h before IL-1β induction (Figure 6A–C). To ensure that the activation of AKT and ERK1/2 is the possible mechanism of action of OXY on inhibiting the IL-1β-induced inflammatory response in HMC3, we performed similar experiments with the presence of the PI3K/AKT and the MAPK inhibitors (LY294002 and U0126, respectively). The cells were harvested to prepare cell lysate for Western blot analysis, and the culture supernatants were also collected for IL-6 and MCP-1 assay by ELISA. Our results showed that LY294002, U0126, or the combination of these two inhibitors could be able to suppress AKT or ERK1/2 phosphorylation (Figure 6D). We found that inhibition of AKT or ERK1/2 alone by LY294002 or U0126 reduced the level of IL-6 and MCP-1 in the culture supernatants of HMC3 cells stimulated with IL-1β (Figure 6E,F). Interestingly, combination of these two inhibitors could significantly decrease the secretion of IL-6 and MCP-1 which was estimated to be an approximately 50% reduction, and these effects were seen to be similar to those of IL-1β-induced HMC3 cells pretreated with OXY (Figure 6E,F). These data strongly suggest that OXY may exert it anti-inflammatory effects in HMC3 cells in response to IL-1β induction mainly by suppressing the activation of AKT and ERK1/2.

**Figure 7:**
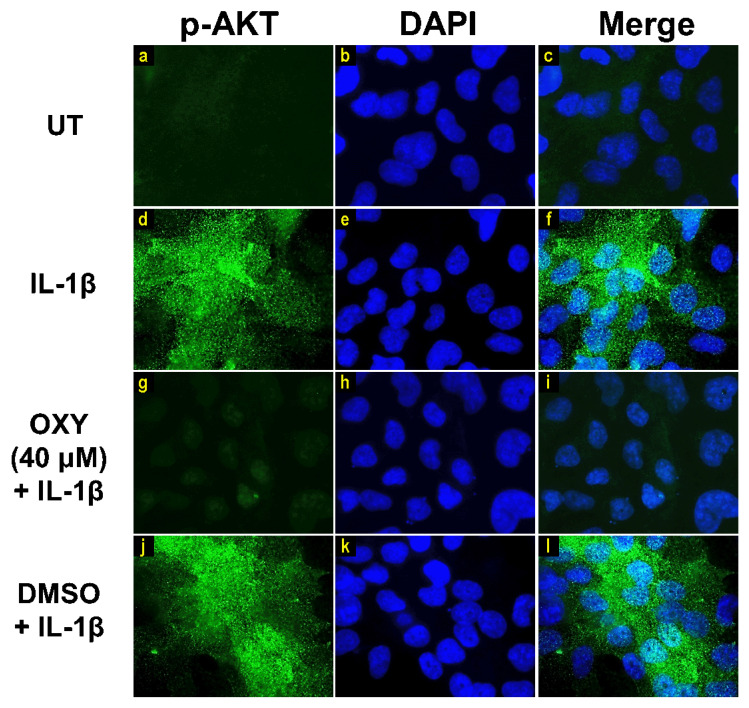
The effects of OXY on regulating AKT phosphorylation in cells stimulated with IL-1β. Representative images from immunofluorescence study showing AKT phosphorylation at Ser473 (green) of untreated cells (**a–c**), IL-1β-stimulated cells (**d–f**), IL-1β-stimulated cells with the presence of OXY at 40 μM (**g–i**), or DMSO (**j–l**). Nuclei (blue) were stained with DAPI. The micrographs were captured at 100× magnification, and data are representatives of three replicates.

**Figure 8:**
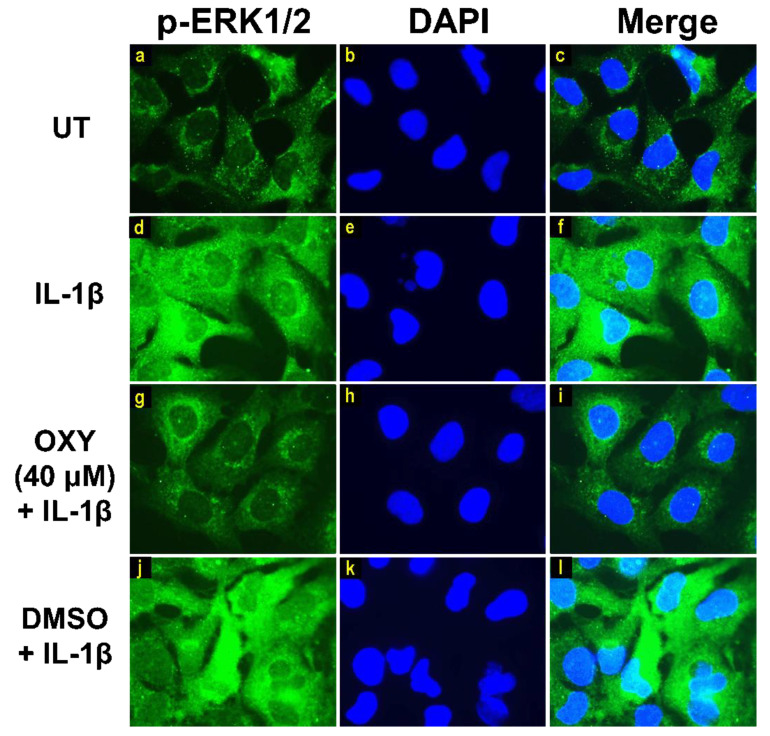
Immunofluorescence study for the effects of OXY on ERK1/2 phosphorylation in response to IL-1β activation in HMC3 cells. Representative images from immunofluorescence study showing ERK1/2 phosphorylation (green) of untreated cells (**a–c**), IL-1β-stimulated cells (**d–f**), IL-1β-stimulated cells with the presence of OXY at 40 μM (**g–i**), or DMSO (**j–l**). Nuclei (blue) were stained with DAPI. The micrographs were captured at 100× magnification, and data are representatives of three replicates.

**Figure 9:**
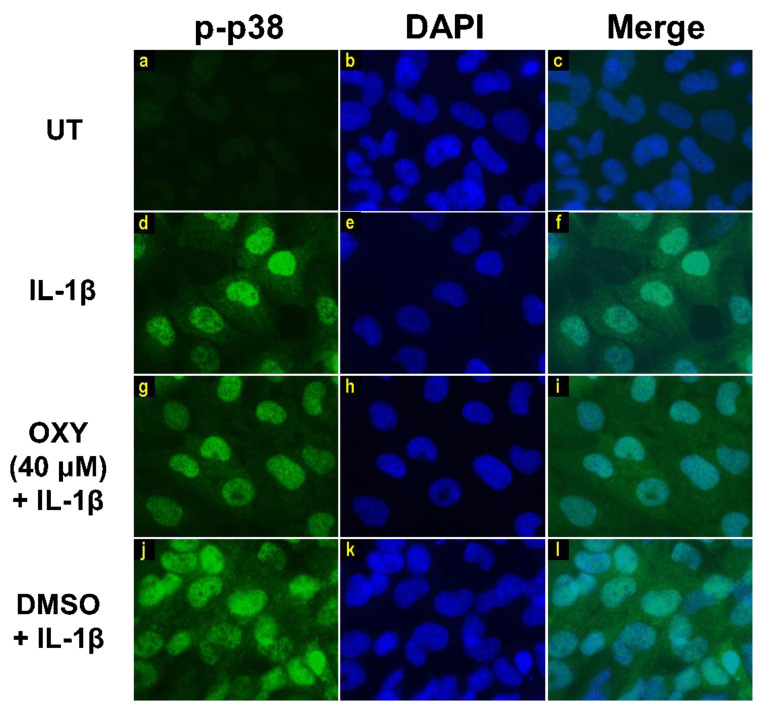
Immunofluorescence study showing the effects of OXY on p38 phosphorylation in response to IL-1β activation in HMC3 cells. Representative images from immunofluorescence study showing p38 phosphorylation (green) of untreated cells (**a–c**), IL-1β-stimulated cells (**d–f**), IL-1β-stimulated cells with the presence of OXY at 40 μM (**g–i**), or DMSO (**j–l**). Nuclei (blue) were stained with DAPI. The micrographs were captured at 100× magnification, and data are representatives of three replicates.

Moreover, the results from immunofluorescence study definitively verified our finding that the phosphorylation of AKT at Ser473 (Figure 7g–i) and ERK1/2 (Figure 8g–i) induced by IL-1β for 20 min were dramatically suppressed by OXY at 40 μM. However, pretreatment with cells with OXY at 40 μM before IL-1β stimulation did not obviously inhibit the phosphorylation of p38 kinase (Figure 9g–i). Altogether, these data revealed that OXY strongly inhibits the phosphorylation of AKT and ERK1/2 at earlier time points.

## 3. Discussion

Microglia residing in the central nervous system (CNS) are necessary as a part of the innate immunity since they respond to injury and infection [18]. Additionally, this type of cell is beneficial to the brain in that they promote neuronal survival and erase cellular debris during the normal neuronal remodeling process, and they can be influential in response to injury and infection [19,20]. However, uncontrollable levels of inflammation in microglia has been proved to be involved in the pathogenesis of neurodegenerative diseases. This overwhelmed inflammation triggered by infection or traumatic stimuli may lead to neurodegeneration and neuronal disruption [18]. IL-1β signaling prominently relates to cellular inflammatory responses in the CNS [21]. Stimulation with IL-1β (as a result of inflammatory exacerbation) causes increased secretion of several different inflammatory mediators, including cytokines and chemokines [7,8]. Therefore, alteration of microglial function has raised a prospective therapeutic scheme [22]. Natural polyphenolic phytochemicals have emerged as possible anti-neuroinflammatory agents. In particular, our previous study demonstrated that the *Artocarpus lakoocha* Roxb, containing a high amount of oxyresveratrol (OXY), could suppress lipopolysaccharide (LPS)-induced inflammatory response via inhibiting PI3K/AKT, MAPK, and NF-κB cascades in murine macrophages [10]. In our current study, we focused mainly on investigating anti-neuroinflammatory effects of OXY, reported to pass the blood–brain barrier (BBB) and exert neuroprotective effects in rodents [12,15,23]. However, the exact mechanisms of OXY in inhibiting the IL-1β-induced inflammatory response in human microglia has not been elucidated. Therefore, we examined the anti-inflammatory effects of OXY in IL-1β-induced HMC3 cells.

Since IL-6 can cause an increase in body temperature, overwhelming of IL-6 levels may cause brain damage, which is a critical condition in patients [24,25]. Furthermore, MCP-1 chemokine plays a major role in a chemotactic process to stimulate and regulate the migration of various cell types, especially in response to inflammation [26]. Interestingly, our current findings showed that secretion of IL-6 and MCP-1 by HMC3 microglia in response to IL-1β exposure could be suppressed by the presence of OXY. Remarkably, in HMC3 cells, IL-1β cannot activate the production of TNF-α and CXCL10 in contrast to another human microglial cell line (C20) which could produce these cytokines in addition to IL-6 and MCP-1 after IL-1β exposure [7]. It is interesting that IL-1β is a more potent inducer of human microglia in comparison to LPS, whereas mouse microglia are extremely sensitive to LPS, but not to IL-1β since they do not express IL-1 receptor (IL-1R) [27]. Although the secretion of pro-inflammatory cytokines was induced by IL-1β, OXY potentially suppressed this effect of IL-1β. This suggests that OXY may be one of the good candidates for the therapy of neuroinflammation. To achieve the understanding at the cellular level, we examined important signal transduction pathways downstream to IL-1R ligation upon IL-1β stimulation. After IL-1R ligation, the upstream signal is relayed by MyD88 binding to the toll/interleukin-1 receptor (TIR) domain and eventually results in the activation of mitogen-activated protein kinases (MAPKs), PI3K/AKT, and nuclear factor-kappa B (NF-κB), which are important in instigating the expression of pro-inflammatory mediators [6,28]. We showed that OXY potently inhibited the phosphorylation of PI3K/AKT and ERK1/2 in HMC3 cells pretreated with OXY for only 4 h. Interestingly, in contrast to MAPK and PI3K/AKT, IL-1β could not solely stimulate NF-κB activation through IκB (an inhibitor of NF-κB) degradation in these human microglia. This effect observed in our current study is consistent with the observations from the previous study which reported that the nuclear translocation of NF-κB was not induced by IL-1β in isolated microglia, but these microglial cells were more energetically induced by IL-1β only when they were co-cultured with astrocytes and/or brain microvessel endothelial cells [21]. These facts exhibit the complexity of cellular signaling in different cell types in the brain where inflammatory response to certain neuronal stimuli requires the synchronization of different specialized cells in the microenvironment. Microglia and astrocytes can crosstalk via secondary messengers including adenosine triphosphate (ATP) and cytokines [29]. One example of this crosstalk is that the cytokines secreted from activated microglia (M1) can activate astrocytes to become the active form (A1) [30]. The activation of these microglia is a result of LPS stimulation [31].

We investigated for the integrity of IκB in HMC3 after IL-1β induction and found that OXY did not have any effect on the degradation status of IκB. These results strongly suggest that OXY may reduce IL-6 and MCP-1 production via suppressing the activation of other signaling molecules. The modulation of ERK1/2 phosphorylation is crucial for relaying the signaling for the production and secretion of different nociceptive factors (cytokines such as TNF-α and IL-1β or enzymes COX-2, iNOS, and nNOS), which are related to an increase in the neuropathic pain sensations [32,33,34]. However, it has been reported that HMC3 cells cannot express iNOS nor produce nitric oxide (NO), even when stimulated with several different inflammatory stimuli [16]. Our current data confirmed the previous report since we could not detect iNOS in this cells in comparison to the high level in the lysate of LPS-induced RAW 264.7 cells. Additionally, the level of NO in the culture supernatant of HMC3 induced with IL-1β was very low. Similarly, we discovered that IL-1β did not induce the production of reactive oxygen species (ROS) in HMC3 cells; it is less likely that the mechanism of OXY in suppressing inflammatory response upon IL-1β stimulation is through the antioxidant effects of OXY.

Like MAPKs, PI3K/AKT signaling also promotes the signal transduction of IL-1R leading to the regulation of inflammatory cytokine gene expression [35,36]. Therefore, we tested the effects of OXY on AKT and ERK1/2 phosphorylation status in HMC3 upon IL-1β stimulation at varied time points. Results showed that OXY could significantly inhibit AKT phosphorylation over the course of IL-1β stimulation, whereas ERK1/2 phosphorylation was moderately suppressed. Specifically, we observed a drastic reduction of AKT phosphorylation (Ser473) and p70S6K, which is a target kinase downstream of PI3K/AKT and normally plays a role in regulating multiple cellular processes, including the neuronal system [37,38]. These results suggest that OXY may have more inhibitory preference toward AKT rather than MAPKs in IL-1β-induced HMC3. Our data obtained from experiments that utilized the PI3K/AKT inhibitor and MEK inhibitor confirmed that the suppression of IL-6 and MCP-1 secretion upon IL-1β stimulation by OXY is truly regulated via its ability to inhibit the IL-1β-induced phosphorylation of AKT and ERK1/2.

**Figure 10:**
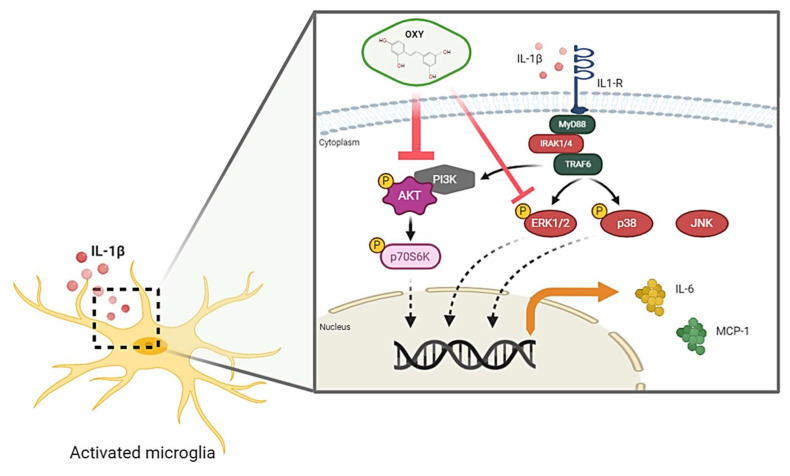
Schematic picture proposing that OXY modulates the IL-1β-stimulated inflammatory response. OXY drastically suppresses the activation PI3K/AKT/p70S6K signal transduction pathway, but mildly inhibits the ERK1/2 MAPK signaling, resulting in the downregulation of pro-inflammatory cytokine and chemokine production in response to IL-1β induction.

In conclusion, the recent study provides the first evidence that OXY possesses great properties to attenuate the IL-1β-activated inflammatory response, mainly through blocking the activation of AKT and ERK1/2 pathways in HMC3 human microglia. The proposed model of the possible mechanism of action of OXY against IL-1β-activated inflammatory response in human microglia, HMC3, is shown in Figure 10. Therefore, our study provides promising data that OXY could be one of the potent agents that may modulate microglial activation and suppress the secretion of pro-inflammatory factors. These properties of OXY make it an interesting candidate to be developed as an anti-neuroinflammation agent.

## 4. Materials and Methods

### 4.1. Oxyresveratrol Preparation

Oxyresveratrol (OXY) with more than 97% purity (HPLC) was obtained (Sigma-Aldrich, Saint Louis, MO, USA) (catalog number 91211). The stock solution of 100 mM was prepared by dissolving the compound in 100% dimethyl sulfoxide (DMSO). The OXY stock solution was aliquoted and stored at −20 °C, in the dark until use. For all experiments, the compound was freshly pre-diluted in serum-free medium to make 1 mM prior to being furthering diluted to final concentrations for each treatment. By using this diluting strategy, it helped enhance the solubility of the compound. For the vehicle control, the final concentration of DMSO was below 0.1% throughout the experiment.

### 4.2. Cell Culture

The Human Microglial Clone 3 (HMC3) cell line (ATCC^®^ CRL-3304 ^TM^) used in this study was purchased from the American Type Culture Collection, (ATCC, Manasssas, VA, USA). The authentication of this cell line can be searched by the ATCC STR database (https://www.atcc.org/STR). The cells were cultured in minimum essential medium (MEM) (Gibco, Thermo Fisher Scientific, Waltham, MA, USA), supplemented with 10% fetal bovine serum (Merck KGaA, Darmstadt, Germany), antibiotics (100 U/mL penicillin and 100 μg/mL streptomycin) (Gibco, Thermo Fisher Scientific, Waltham, MA, USA), 1× MEM non-essential amino acids (Gibco, Thermo Fisher Scientific, Waltham, MA, USA), and 1 mM sodium pyruvate (Gibco, Thermo Fisher Scientific, Waltham, MA, USA), and they were maintained under a humidified atmosphere of 37 °C, 5% CO_2_. The cells were sub-cultured when they reached 80–90% confluence. We used HMC3 at passage (P.) 2 to 10. The passage number of HMC3 used in this study was not allowed to exceed P.10. All experimental procedures were approved by the institutional biosafety committee (IBC). The IBC approval number is CMUIBC02002/2563, and the date of approval is 12 March 2020.

### 4.3. Cell Viability Assay

Cell viability was assayed by 3-(4,5-dimethylthiazol-2-yl)-2,5-diphenyltetrazolium bromide (MTT) to differentiate the range of toxic and nontoxic concentrations following the previously published protocol [39]. HMC3 cells were seeded in 96-well plates at a density of 1 × 10^4^ cells per well for 24 h. Cells were incubated with OXY at varied concentrations ranging from 0.625 to 640 μM or with vehicle (DMSO at 0.0002–0.16%) for 48 h. MTT reagent (0.4 mg/mL in PBS) was added to each well, and cells were incubated at 37 °C, 5% CO_2_ for 1 h. The culture supernatants were discarded, and 200 μL of DMSO was added to each well. The absorbance at 570 nm was measured using a microplate reader (BioTek Instruments, Winooski, VT, USA). MTT assay was performed 3 times, and each assay was undertaken in triplicate (*n* = 3). The half maximal inhibitory concentration (IC_50_) value and the range of non-cytotoxic and cytotoxic concentrations of OXY to the cells were obtained. Three different non-cytotoxic concentrations (10, 20, and 40 μM) were selected for all experiments.

### 4.4. Enzyme-Linked Immunosorbent Assay (ELISA)

HMC3 cells were treated with differential concentrations of OXY with the presence of IL-1β for 24 h. The culture supernatants of the cells were collected for cytokine detection. The secreted amount of IL-6, MCP-1, TNF-α, and CXCL10 in the culture supernatants was measured by using an ELISA kit (MAX^TM^ Deluxe Set) for each cytokine or chemokine (BioLegend, San Diego, CA, USA) following the manufacturer’s protocol. Briefly, the capture antibody was coated into individual wells of immunoplate and incubated at 4 °C overnight. The plate was then blocked with blocking buffer for 1 h at room temperature (RT). Sample culture supernatants were added into each well, and plates were incubated at RT for 2 h. A detection antibody was then added and incubated for 1 h. After washing, the plate was incubated with an avidin–HRP solution for 30 min. Then TMB substrate solution was added, and a stop solution was applied to each well. The absorbance at 450 and 570 nm was read with a microplate reader (BioTek Instruments, Winooski, VT, USA). For some experiments that aimed to explore the involvement of AKT and ERK1/2 activation in response to IL-1β induction, a specific inhibitor of the PI3K/AKT (LY294002) and a MEK inhibitor (U0126) were used.

### 4.5. Western Blot Analysis

For examining the effects of OXY on inhibiting IL-1β-stimulated phosphorylation of crucial proteins in PI3K/AKT, MAPK, and NF-κB signaling pathways. The OXY-treated HMC3 cells and stimulated with IL-1β at suitable time points were collected for cell lysate preparation. In some experiments, the potency of OXY on growth and survival signaling were tested by inducing cells with IL-1β at increasing concentrations (1, 10, and 100 ng/mL) for 20 min. The cell lysates were heated at 100 °C for 5 min, separated by SDS–PAGE, and transferred onto polyvinylidene difluoride (PVDF) membranes (GE Healthcare Life Science, Marlborough, MA, USA). After blocked with 5% skim milk in a mixture of tris-buffered saline and Tween 20 (TBS-T) (0.02 M Tris-HCl, pH 7.6, 0.0137 M NaCl, and 0.05% Tween 20) (all reagents from Sigma-Aldrich, Saint Louis, MO, USA) at RT for 1 h, membranes were incubated with an appropriate primary antibody (Cell Signaling Technology, Boston, MA, USA) at 4 °C for 24 h. Primary antibodies used in this study included an anti-NF-κB p65 (D14E12) antibody (catalog number 8242), a mouse anti-IκBα (L35A5) antibody (catalog number 4814), a phosphospecific rabbit anti-p44/42 MAPK (Erk1/2) (Thr202/Tyr204) antibody (catalog number 4370), a mouse anti-p44/42 MAPK (Erk1/2) antibody (catalog number 9107), a phosphospecific mouse anti-p38 MAPK (Thr180/Tyr182) antibody (catalog number 9216), a rabbit anti-p38 MAPK (D13E1) antibody (catalog number 8690), a phosphospecific rabbit anti-SAPK/JNK (Thr183/Tyr185) antibody (catalog number 4668), a rabbit anti-SAPK/JNK antibody (catalog number 9252), a phosphospecific rabbit anti-AKT (Ser473) antibody (catalog number 4060), a mouse anti-AKT (40D4) antibody (catalog number 2920), a phosphospecific rabbit p70 S6 kinase (Thr389) antibody (catalog number 97596), or a mouse anti-β-actin antibody (catalog number MA1115) (Boster Biological Technology, Pleasanton, CA, USA). Membranes were washed three times with TBS-T and incubated with secondary antibodies (LI-COR Biosciences, Lincoln, NE, USA), which included 1:10,000 of an anti-mouse IgG conjugated with IRDye^®^800CW (catalog number 926-32210) or 1:10,000 an anti-rabbit IgG conjugated with IRDye^®^680RT (catalog number 926-68071) at RT for 2 h. In some experiments where we wanted to determine whether OXY suppresses the production of cytokines via inhibiting AKT and ERK1/2 phosphorylation, a specific inhibitor of the PI3K/AKT (LY294002) and a MEK inhibitor (U0126) (both at 10 µM) were added to the cells 1 h prior to IL-1β stimulation. The reactive signal of each protein were detected by an Odyssey^®^ CLx Imaging System (LI-COR Biosciences, Lincoln, NE, USA). ImageJ software (developed at the National Institutes of Health, USA, http://rsb.info.nih.gov/ij).was used to analyze immunoreactive bands of each protein.

### 4.6. Immunofluorescence Study

To visualize the phosphorylation status of AKT and MAPKs, including ERK1/2 and p38, upon IL-1β activation, immunofluorescence was performed. HMC3 cells were seeded on glass cover slips and treated with OXY for 4 h before induced with 1 ng/mL of IL-1β at appropriate time points. After treatment and stimulation, cells were fixed with 4% paraformaldehyde/PBS (Sigma-Aldrich, Saint Louis, MO, USA) for 15 min at RT. Cells were then washed three times with PBS (5 min each). Cells were then permeabilized with 0.3% Triton X-100 in PBS for 5 min. The cells were then blocked with 1% BSA/PBS for 1 h. For p-ERK1/2, cells were fixed and permeabilized with ice-cold 100% methanol for 15 min on ice. The sample coverslips were incubated with appropriate antibodies (Cell Signaling Technology, Boston, MA, USA) diluted in 1% BSA/PBS at 4 °C overnight. Primary antibodies included 1:400 of a phosphospecific rabbit anti-AKT (Ser473) antibody (catalog number 4060), 1:200 of a phosphospecific rabbit anti-p44/42 MAPK (Erk1/2) (Thr202/Tyr204) antibody (catalog number 4370), and 1:200 of a phosphospecific mouse anti-p38 MAPK (Thr180/Tyr182) antibody (catalog number 9216) (1:200). Secondary antibodies were used at a 1:500 dilution. They included Alexa488-conjugated anti-rabbit IgG or Alexa594-conjugated goat anti-rabbit IgG (Thermo Fisher Scientific, Waltham (HQ), MA, USA). In addition, the nuclei were stained with 1 μg/mL of 4′,6-diamidino-2-phenylindole (DAPI) (Sigma-Aldrich, Saint Louis, MO, USA) for 2 h, in the dark at RT. After washing three time with PBS (5 min each) and one time with distilled water (5 min), sample cover slips were mounted with Fluoromount-G (SouthernBiotech, Birmingham, AL, USA). The observations were performed on a fluorescence microscope, Axio Vert.A1 (Carl Zeiss, Oberkochen, Germany), with 100x magnification, and micrographs were captured with the Zen2.6 (blue edition) Software for the Zeiss Axiocam 506 color microscope camera.

### 4.7. Statistical Analysis

All data from the experiments were represented as mean ± SD and then analyzed by one-way analysis of variance (ANOVA) with Tukey’s post hoc multiple comparisons on raw data reads using SPSS software (SPSS Inc., Chicago, IL, USA). A *p*-value (*p* < 0.05) was considered statistically significant.

## Supplementary Materials

Supplementary materials can be found at: https://www.mdpi.com/1422-0067/21/17/6054/s1.

## Abbreviations

CNS	Central nervous system
ELISA	Enzyme-linked immunosorbent assay
HMC3	Human microglia clone 3
IL-1β	Interleukin-1 beta
IL-1R	Interleukin-1 receptor
IL-6	Interleukin-6
MAPK	Mitogen-activated protein kinase
MCP-1	Monocyte chemoattractant protein-1
NF-κB	Nuclear factor-kappa B
OXY	Oxyresveratrol
TNF-α	Tumor necrosis factor-alpha
