# Supplementary material for: Oxyresveratrol Inhibits IL-1β-Induced Inflammation via Suppressing AKT and ERK1/2 Activation in Human Microglia, HMC3
Source: Int J Mol Sci. 2020 Aug 22;21(17):6054. doi: 10.3390/ijms21176054 (PMC7504001; doi:10.3390/ijms21176054)
Supplement: Supplementary file 1 [file ijms-21-06054-s001.pdf]

Supplementary Information

1. Materials and Methods

1.1. Nitric Oxide (NO) Production Assay

It has been reported that HMC3 cells could not produce the NO level caused by the unexpressive enzyme of inducible nitric oxide synthase (iNOS) after the stimulation of various pro-inflammatory exposures, such as lipopolysaccharide (LPS), LPS/ interferon gamma (IFNγ), IL-1β, and TNF-α [1]. To ensure that NO is not secreted in response to IL-1β activation in this particular cell line, cells were plated in a 24-well plate for 24 h followed by pretreatment with OXY (10, 20, and 40 μM) 4 h prior to IL-1β (1 ng/mL) induction for 24 h. The mouse RAW 264.7 macrophages, which generally produce NO, were exposed to LPS (1 μg/mL) for 24 h to serve as a positive control. We performed Western blot analysis to confirm whether IL-1β induces iNOS expression, which is a responsible enzyme for NO production. The cell lysates were separated by SDS–PAGE and transferred onto polyvinylidene difluoride (PVDF) membranes (GE Healthcare Life Science, Marlborough, MA, USA). After being blocked with 5% skim milk at RT for 1 h, membranes were incubated with a rabbit anti-iNOS (EPR16635) antibody (catalog number ab178945) (Abcam plc, Cambridge, UK) or a mouse anti-β-actin antibody (catalog number MA1115) (Boster Biological Technology, Pleasanton, CA, USA). Subsequently, the membranes were incubated with anti-rabbit/mouse IgG conjugated with IRDye® secondary antibodies at RT for 2 h. The reactive signal of each band was detected by an Odyssey® CLx Imaging System (LI-COR Biosciences, Lincoln, NE, USA).

Furthermore, the presence of nitrite concentration in 24 h-conditioned media was quantified as an indicator of NO production using Griess' reagent (Sigma-Aldrich, Saint Louis, MO, USA). Briefly, 50 μL of culture supernatant was mixed with 50 μL of Griess reagent for 15 min to yield nitrite, detected as a pink color. The absorbance was detected and quantified using a microplate reader (BioTek Instruments, Winooski, VT, USA) at 540 nm and then assessed based on a nitrite standard curve.

1.2. Reactive Oxygen Species (ROS) Measurement

The production of ROS in the cell was detected by using a cell permeable fluorescent dye 2,7'-dichlorofluorescein diacetate (DCFH-DA) (Sigma-Aldrich, Saint Louis, MO, USA) [2]. HMC3 cells cultured in 96-well plates were pretreated with OXY at 10, 20, and 40 μg/mL for 1 h, and then the cells were stimulated with 1 ng/mL of IL-1β or 500 μM of hydrogen peroxide (H_2O_2) for 1 h. For ROS detection, the treated cells were incubated at 37 °C for 30 min in serum-free media and 20 μM DCFH-DA. The fluorescence intensity was detected with a microplate reader (BioTek Instruments, Winooski, VT, USA) with an excitation wavelength at 485 nm and an emission wavelength of 535 nm. Additionally, cells were washed with PBS 3 times, and the fluorescent signal was visualized by a fluorescent microscope. Micrographs were captured with Zen2.6 (blue edition) software for the Zeiss Axiocam 506 color microscope camera.

2. Results

2.1. The Level of Nitric Oxide (NO) Was Not Detectable in IL-1β-Stimulated HMC3 Cells, and OXY Did Not Affect NO Production

To investigate the expression of iNOS protein in IL-1β-induced HMC3 cells, Western blot analysis was used. Exposure of IL-1β in human HMC3 microglia could not apparently alter iNOS expression when compared to a significantly high expression level of iNOS ($p < 0.05$, Figure S1A–D) in the LPS-induced RAW 264.7 macrophages (which were used as a positive control). OXY did not affect iNOS protein expression (Figure S1A,C). We further performed NO detection by using Griess reagent to confirm that IL-1β does not activate the production of NO in HMC3 cells. As expected, the results clearly showed that IL-1β addition did not induce the production of nitrite in HMC3 microglia, and OXY treatment did not have any effect on the NO level (Figure S1E,F). However, compared to RAW 264.7 macrophages, LPS strongly stimulated the nitrite production by 8-fold (Figure S1E,G). Therefore, these results evidently indicate that nitric oxide is not produced in response to IL-1β activation in HMC3 cells due to the fact that there is no iNOS protein expression in this cell line.

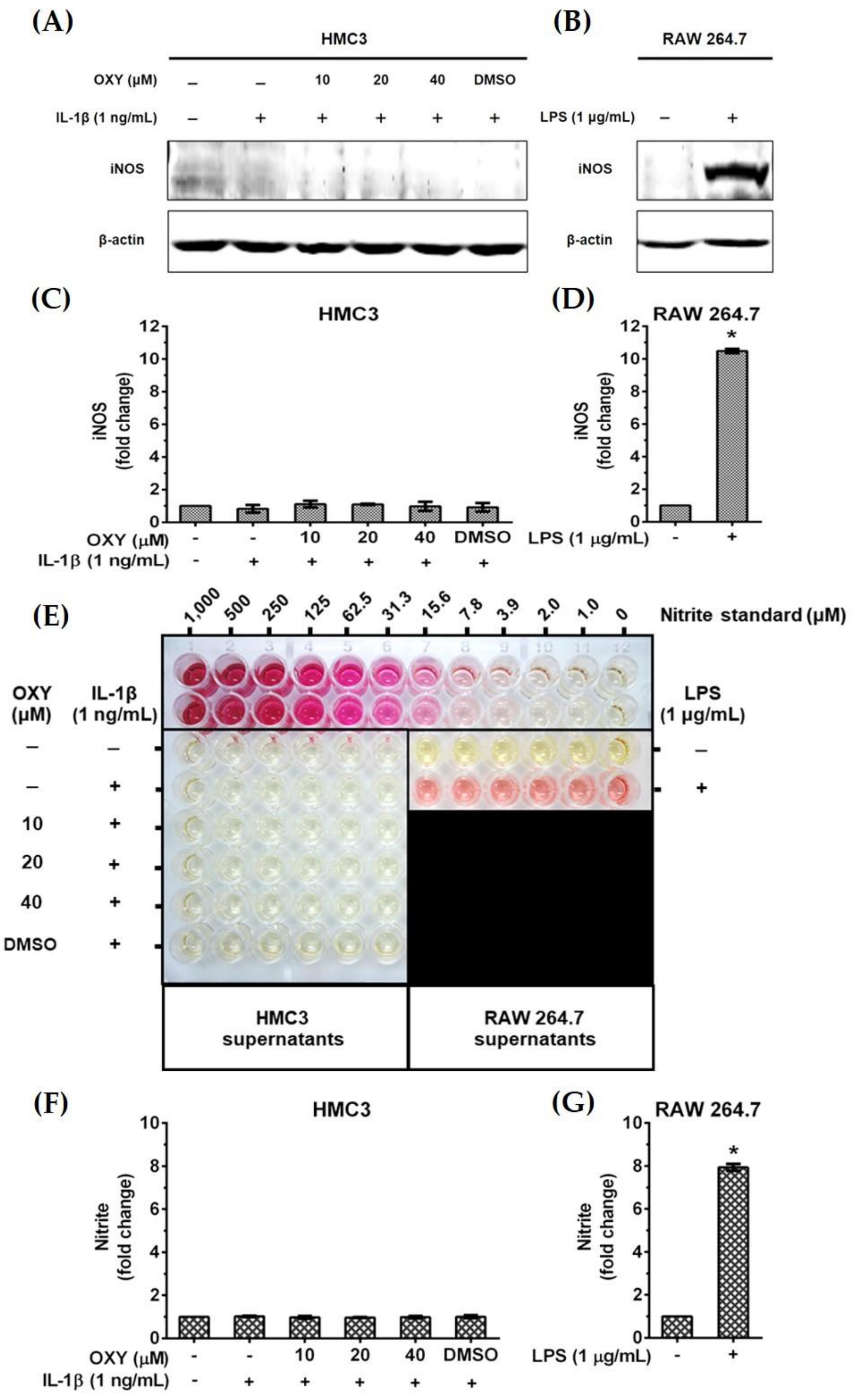

Figure S1. Effects of OXY on NO production in IL-1β-induced HMC3 microglia. **(A)** the effects of OXY on the expression level of iNOS in HMC3 cells after IL-1β activation for 24 h; **(B)** the expression level of iNOS in RAW 264.7 macrophages after LPS induction for 24 h (positive control); **(C,D)**

quantitative analysis of iNOS protein expression; β-actin was detected and used as an internal control; **(E)** the image showed the presence of nitrite forming a pink diazo dye in IL-1β-stimulated HMC3 supernatants and LPS-induced RAW 264.7 supernatants; **(F,G)** quantitative analysis of the production of nitrite. Data are represented as mean ± SD of independent experiments; $^{*}p < 0.05$ (compared to the untreated group).

2.2. IL-1β Did Not Induce The Production of ROS, and OXY Potently Reduced Intracellular ROS Level in H_2O_2-Induced HMC3 Cells

We performed additional experiments to verify whether IL-1β induces ROS production in HMC3 cells and OXY reduces this event as a major mechanism of inhibiting inflammatory response. Results clearly showed that IL-1β did not significantly induce ROS production in HMC3 cells (Figure S2Ab). We designed an experiment to confirm that this particular microglial cell line can generate intracellular production in response to hydrogen peroxide. We found that H_2O_2 strongly induced ROS production in each individual HMC3 cell, but OXY at all concentrations used potently inhibited intracellular ROS in these cells (Figure S2A,B).

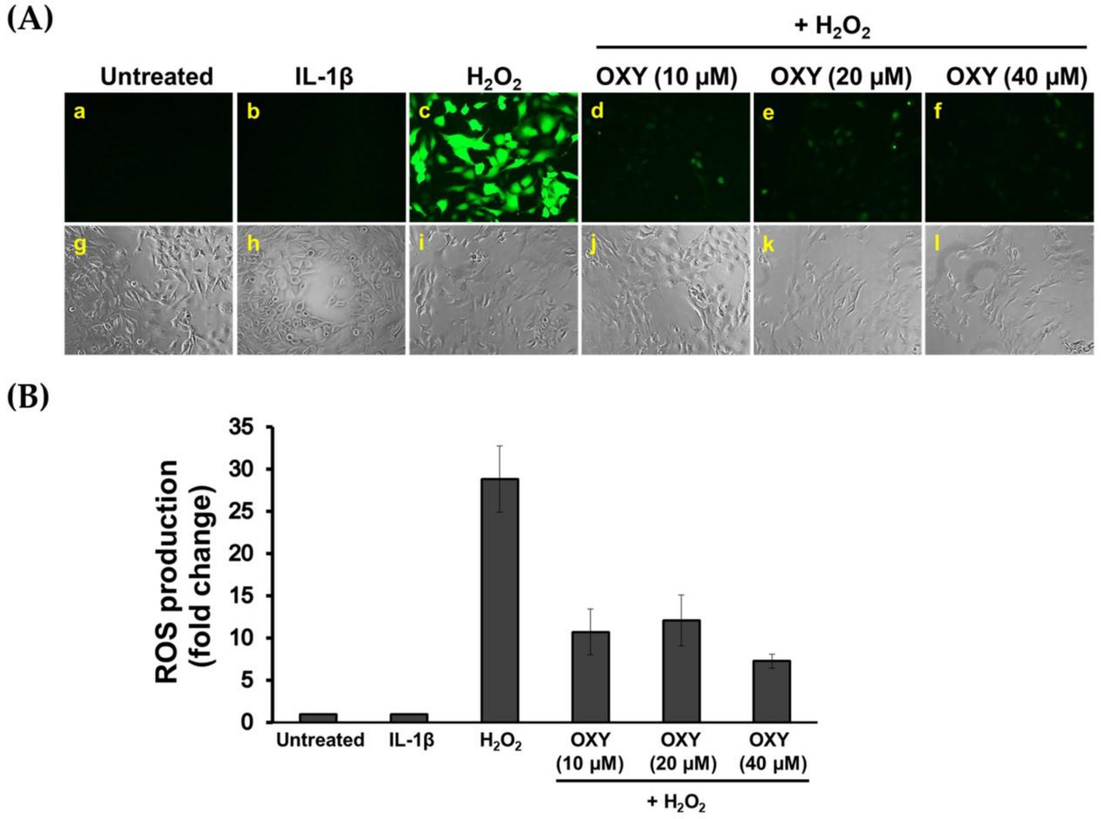

Figure S2. ROS measurement in HMC3 cells induced with IL-1β or hydrogen peroxide (H_2O_2) with the presence of OXY at different concentrations. **(A)** Fluorescence staining images of intracellular ROS and **(B)** quantitative analysis of ROS level in HMC3 cells. Data are represented as mean ± SD of independent experiments; $^{*}p < 0.05$ (compared to the untreated group).

References

1. Dello Russo, C.; Cappoli, N.; Coletta, I.; Mezzogori, D.; Paciello, F.; Pozzoli, G.; Navarra, P.; Battaglia, A., The human microglial HMC3 cell line: where do we stand? A systematic literature review. *J. Neuroinflammation* **2018,** 15, (1), 259.
2. Shafiekhani, M.; Ommati, M. M.; Azarpira, N.; Heidari, R.; Salarian, A. A., Glycine supplementation mitigates lead-induced renal injury in mice. *J Exp Pharmacol* **2019,** 11, 15-22.
